# Regulatory Role of N6-methyladenosine (m^6^A) Modification in Osteosarcoma

**DOI:** 10.3389/fonc.2021.683768

**Published:** 2021-05-19

**Authors:** Yujie Zhang, Yanyan Wang, Liwei Ying, Sifeng Tao, Mingmin Shi, Peng Lin, Yangxin Wang, Bin Han

**Affiliations:** ^1^ Department of Orthopedic Surgery, The Second Affiliated Hospital, Zhejiang University School of Medicine, Hangzhou, China; ^2^ Department of Oncology Surgery, The Second Affiliated Hospital, Zhejiang University School of Medicine, Hangzhou, China

**Keywords:** N6-methyladenosine (m^6^A), molecular mechanisms, osteosarcoma, biomarker, therapeutic target

## Abstract

Osteosarcoma is the most common primary bone malignancy, typically occurring in childhood or adolescence. Unfortunately, the clinical outcomes of patients with osteosarcoma are usually poor because of the aggressive nature of this disease and few treatment advances in the past four decades. N6-methyladenosine (m^6^A) is one of the most extensive forms of RNA modification in eukaryotes found both in coding and non-coding RNAs. Accumulating evidence suggests that m^6^A-related factors are dysregulated in multiple osteosarcoma processes. In this review, we highlight m^6^A modification implicated in osteosarcoma, describing its pathophysiological role and molecular mechanism, as well as future research trends and potential clinical application in osteosarcoma.

## Introduction

Osteosarcoma is a relatively rare bone malignancy that predominantly occurs in children and adolescents (1, 2). It is highly aggressive and difficult-to-treat (3, 4). Although the introduction of chemotherapy since the 1970s has increased the 5-year survival rate of patients with non-metastatic osteosarcoma to 70%, the 5-year survival rate of patients with metastatic osteosarcoma is only 20% (5, 6). More importantly, metastasis of osteosarcoma is not uncommon (7). Osteosarcoma is known to exhibit high heterogeneity and significant genome complexity (8–10); thus, tremendous efforts are needed to define the biology of osteosarcoma for developing new therapeutic alternatives.

The discovery of heritable alterations of chromatin structure and DNA modifications that do not change their DNA or RNA sequence itself pioneers a new field of epigenetics (11, 12). N6-methyladenosine (m^6^A) modification refers to the addition or deletion of the methyl group to/from the nitrogen on the 6th carbon of the adenine nucleotide, which is one of the most abundant epigenetic modifications found in RNA molecules (13). First discovered by Desrosiers in the 1970s, m^6^A modification has been found to be involved in almost all steps of RNA processing and metabolism, including pre-mRNA splicing, export, translation, stabilization, and degradation (14–19). With the burgeoning advances in molecular biology and sequencing, m^6^A modification has been reported to be implicated in virtually all cellular functions and multiple diseases (including osteosarcoma) (20–23). In this review, we intended to summarize the current advances in the pathophysiological roles and molecular mechanism of m^6^A modification in osteosarcoma and related diseases, and discuss the potential clinical application of m^6^A modification in osteosarcoma.

## m^6^A Regulation

**Figure 1:**
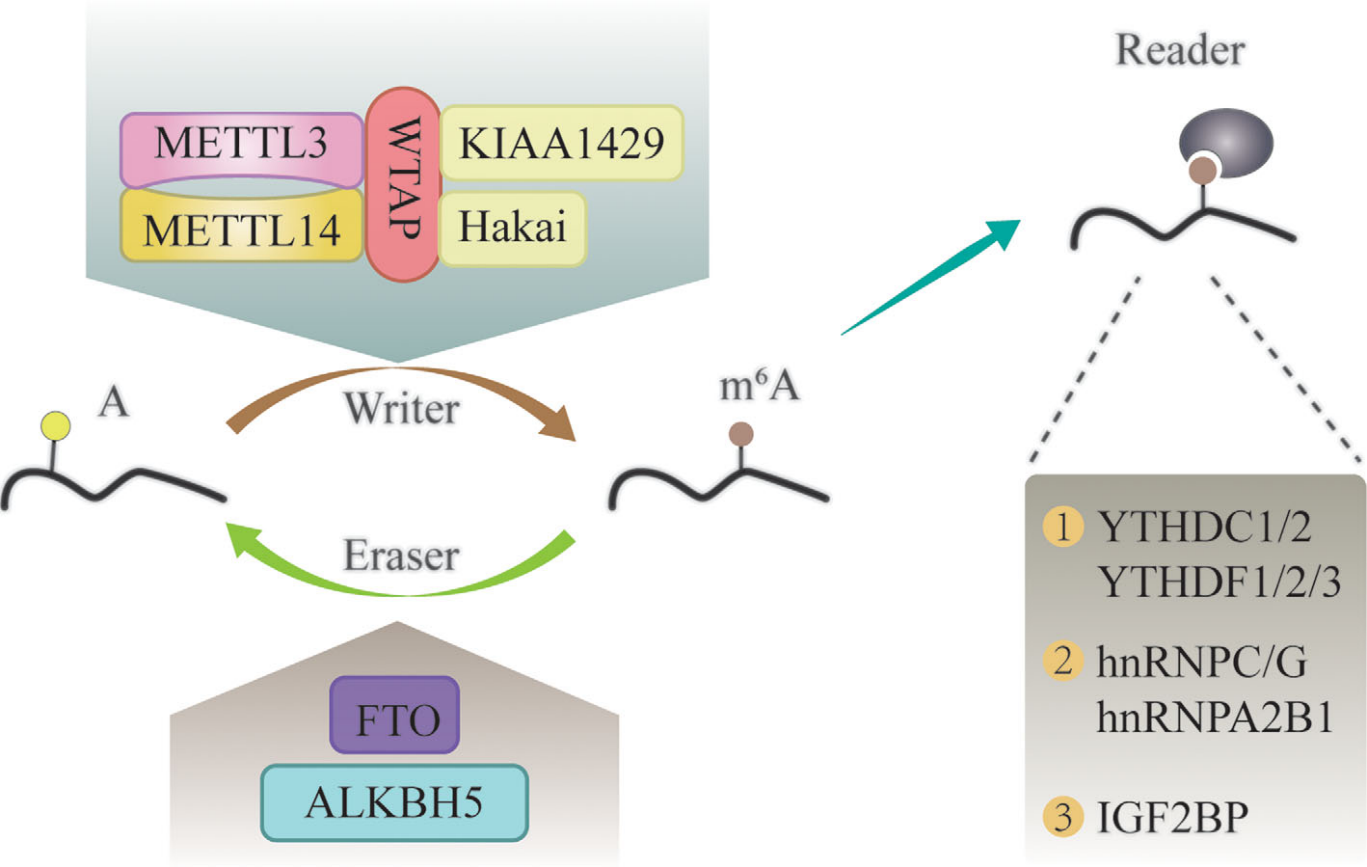
The molecular mechanism of m^6^A modification. It is a dynamic and reversible epigenetic modification that is regulated by “writers” and “erasers.” m^6^A markers in the RNA can be recognized by “readers”.

Similar to DNA and histone methylation, m^6^A modification is a dynamic and reversible process that is regulated by methylases and demethylases (**Figure 1**) (24, 25).

M^6^A methylases, also called “writers”, can transfer a methyl group to the N-6 position of adenosine in the nucleic acid (26). It commonly works in the form of a multicomponent m^6^A methyltransferase complex (MTC) (27). Methyltransferase-like 3 (METTL3) has long been considered as the central catalytic subunit of MTC, while other components have recently been discovered (28). METTL14 is an allosteric adaptor of METTL3 that can stabilize METTL3 and recognize the substrate (29). In addition, Wilms tumor 1-associated protein (WTAP), Vir-like m^6^A methyltransferase associated (KIAA1429), and Cbl proto-oncogene-like 1 (Hakai) participate in the formation of MTC and play a vital role in m^6^A modification (30–32).

M^6^A demethylases, also named “erasers”, are proteins that remove the methyl groups from RNA; thus, conferring a reversible and dynamic nature to the regulation of m^6^A methylation (33). These enzymes mainly include fat mass and obesity-associated protein (FTO), and α-ketoglutarate-dependent dioxygenase alk B homolog 5 (ALKBH5) (34, 35). FTO was the first identified m^6^A demethylase (36). Since then, research on m^6^A modification has gained attention. FTO can demethylate m^6^A into N6-hydroxymethyladeosine (hm^6^A), which is further converted to N6-formyladenosine (f6A) before being hydrolyzed to the stable form adenine (37). The writers and erasers maintain the dynamic balance of m^6^A in the transcriptome, which is essential for normal physiological processes.

In addition to writers and erasers, “readers” are binding proteins that recognize the chemical signatures that are important for the regulation of m^6^A modification (38). The YTH domain-containing proteins are the first discovered readers, which interact with m^6^A through a “tryptophan cage” (39). The family of YTH domain-containing proteins consist of the following five proteins: YTHDC1, YTHDC2, YTHDF1, YTHDF2, and YTHDF3 (40). M^6^A can alter the structure of RNA substrates, and thereby, promote the binding of RNA-binding proteins (RBPs) to substrates (41). These RBPs are called “m^6^A structural switch” readers, and include heterogeneous nuclear ribonucleoprotein C (HNRNPC), HNRNPG, and HNRNPA2B1 (25, 41, 42). Insulin-like growth factor 2 mRNA-binding (IGF2BP) proteins (IGF2BP1, IGF2BP2, and IGF2BP3) are another category of m^6^A readers, which can stabilize RNA by interacting with YTHDF proteins or in an m^6^A structural switch manner (43, 44).

**Figure 2:**
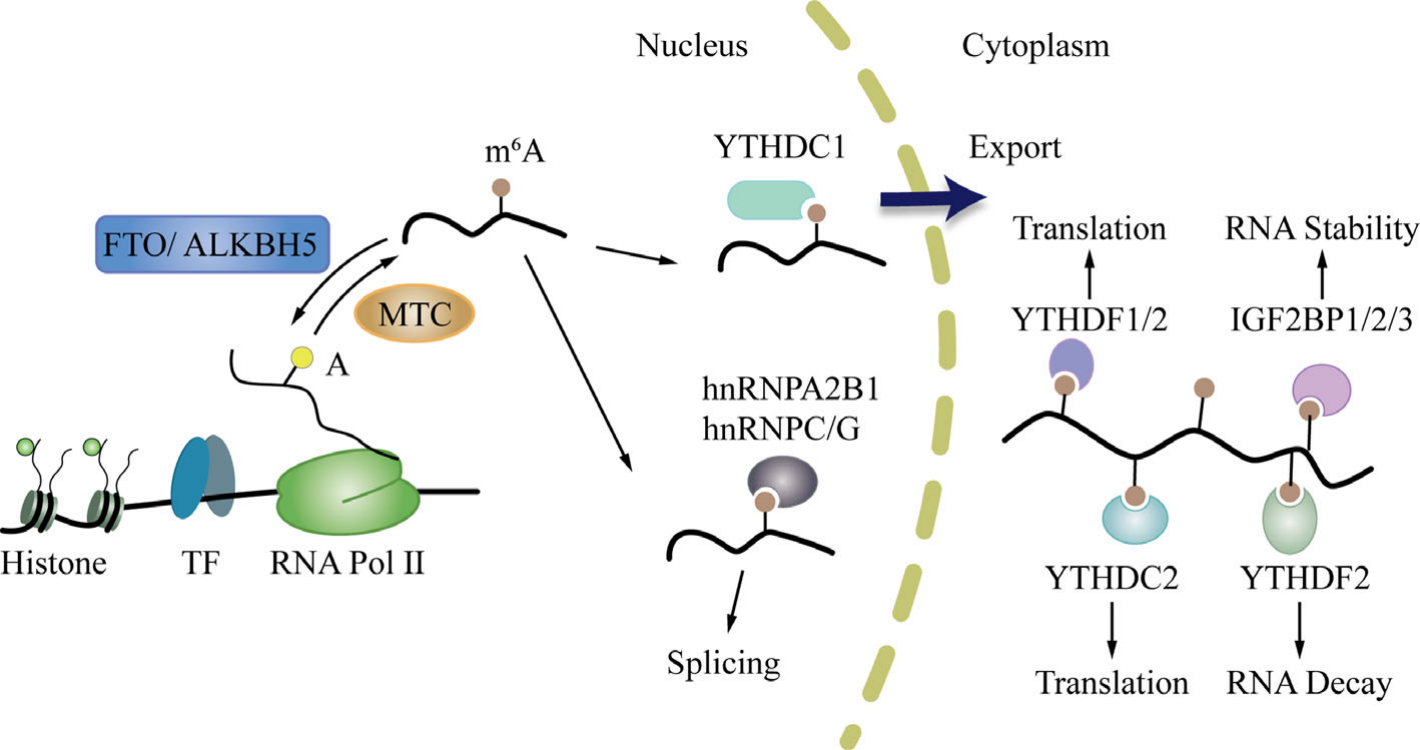
The life cycle of m^6^A mRNA. First, m^6^A writers and erasers regulate the change of m^6^A during transcription in the nucleus. After that, m^6^A can bind to specific nuclear readers, and influence mRNA splicing, exporting, and other bioprocesses. Then, m^6^A is exported to the cytoplasm where it binds to cytoplasm readers and influences mRNA decay, translation, and stabilization.

M^6^A can affect the whole process of gene expression, including transcription, post-transcription, translation, and post-translation (45). In the “life cycle” of m^6^A mRNA (**Figure 2**) (46, 47), the m^6^A writers and erasers first regulate the change in m^6^A during transcription in the nucleus. Then, m^6^A binds to specific nuclear readers and affect mRNA splicing, exporting, and other bioprocesses (48, 49). After being exported to the cytoplasm, the cytoplasm readers bind to m^6^A and influence mRNA decay, translation, and stabilization (46, 50). Through these processes, m^6^A modification regulates gene expression and exerts profound and diverse functions in cell differentiation, immune response, and disease development, especially tumorigenesis, progression, and cancer metastasis (51–54).

## m^6^A and Bone Development

The bone is a complex connective tissue, which is always under a dynamic balance between bone formation mediated by osteoblasts and bone resorption regulated by osteoclasts (55). If the bone homeostasis is disturbed, many bone metabolic diseases such as osteosarcoma, osteoporosis, and osteoarthritis may occur (56). M^6^A is reported to participate in the regulation of bone homeostasis (57). METTL3 is significantly increased during the process of osteogenic differentiation of the bone marrow stem cells (BMSCs). Specifically, silencing METTL3 can suppress the osteogenic differentiation by directly and indirectly regulating RUNX2 (METTL3/m^6^A-pre-miR-320/miR-320-RUNX2 Axis) in BMSCs (58). Remarkably, the expression of METTL3 was also increased during osteoclast differentiation. It can regulate the bone resorption by controlling Atp6v0d2 mRNA degradation and Traf6 mRNA nuclear export (59). METTL14 was found to be positively associated with bone formation in older women with fractures and ovariectomized mice. It promotes osteoblast activity by regulating miR-103-3p processing *via* microprocessor protein DGCR8 (60). Similarly, the FTO, represented as the RNA demethylase, is also closely related to the fate of BMSCs. Li et al. (61) reported that FTO could interact with miR-149-3p and promote osteogenic differentiation of BMSCs.

## m^6^A and Osteosarcoma

Osteosarcoma always occurs where the bones are growing the fasted (62). The inseparable relationship between m^6^A regulators and bone development implies that m^6^A modification might contribute to the progression of osteosarcoma. Herein, we comprehensively review the current research on the association of m^6^A and osteosarcoma.

### Dysregulation of m^6^A Writers in Osteosarcoma

In osteosarcoma cells, m^6^A writers, including METTL3, METTL14, WTAP, and KIAA1429 are mostly present in the nucleus (63). Zhou et al. (64) revealed that METTL3 acts as an oncogene in osteosarcoma. Knockdown of METTL3 can suppress the proliferation, migration, and invasion of the human osteosarcoma cell lines SAOS-2 and MG63 by inhibiting the m^6^A methylation level and expression of the ATPase family AAA domain-containing protein 2 (ATAD2). Additionally, Miao et al. (65) found that METTL3 expression and m^6^A methylation levels were higher in human osteosarcoma tissues and osteosarcoma cells. METTL3 can promote osteosarcoma development by directly increasing the m^6^A methylation level and the expression of lymphoid enhancer-binding factor 1(LEF1), and by activating the Wnt/β-catenin signaling pathway. METTL3 has also been found to facilitate the methylation of GTP-binding protein (DRG) 1, and thereby, promoting osteosarcoma growth, migration, and colony formation (66). Conversely, METTL14 has an inhibitory effect in osteosarcoma. METTL14 overexpression promotes osteosarcoma cell apoptosis and slows tumor progression through caspase 3 activation (67). Recently, Chen et al. (68) demonstrated that another m^6^A writer, WTAP, can positively regulate osteosarcoma tumorigenesis and metastasis by reducing the stability of HMBOX1 in a m^6^A-dependent manner. Inhibiting PI3K/AKT pathways can partly reverse WTAP/HMBOX1- induced osteosarcoma progression.

**Figure 3:**
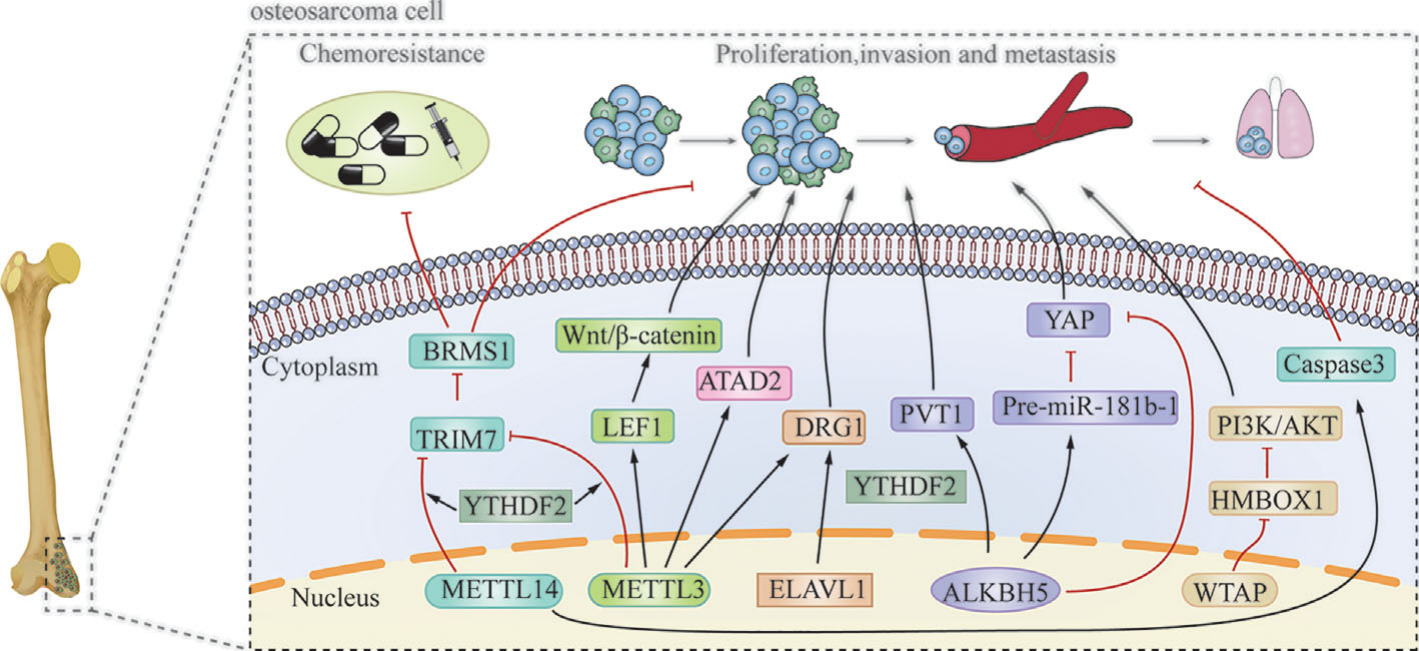
The pathophysiological roles and molecular mechanism of m^6^A modification in osteosarcoma.

METTL3/14 also plays an important role in modulating the chemoresistance of osteosarcoma. METTL3 and METTL14 decrease the RNA level of tripartite motif 7 (TRIM7), while the upregulation of TRIM7 can increase metastasis and the chemoresistance of osteosarcoma by regulating ubiquitination of breast cancer metastasis suppressor 1 (BRMS1) (69). The transcriptome-wide m^6^A sequencing result of chemoresistant osteosarcoma stem cells revealed that over-expression of METTL3 and low METTL14 expression are associated with doxorubicin chemoresistance and stemness of osteosarcoma cells (70). The regulatory mechanism of writer is shown in **Table 1** and **Figure 3**.

**Table 1:** Roles of m^6^A regulators in osteosarcoma.

M^6^A regulators	Role in cancer	Biological function	Target/signaling axis	Ref.
METTL3	Oncogene	Promote cell proliferation, migration, and invasion	ATAD2	(64)
		Promote cell proliferation, migration, and invasion	LEF1/Wnt/β-catenin	(65)
		Promote osteosarcoma growth, migration, and colony formation	DRG1	(66)
	Tumor suppressor	Suppress osteosarcoma metastasis and chemoresistance	TRIM7/BRMS1	(69)
METTL14	Tumor suppressor	Promotes osteosarcoma cell apoptosis and slows tumor progression	Caspase 3	(67)
		Suppress osteosarcoma metastasis and chemoresistance	TRIM7/BRMS1	(69)
WTAP	Oncogene	Promote proliferation and metastasis	HMBOX1/PI3K/AKT	(68)
ALKBH5	Oncogene	Promote cell proliferation, tumor growth	PVT1	(71)
	Tumor suppressor	Suppress cell proliferation, tumor growth	Pre-miR-181b-1/YAP	(72)
YTHDF2	Oncogene	Promote cell proliferation, tumor growth	Critical for ALKBH5-mediated PVT1 stability	(71)
	Tumor suppressor	Suppress osteosarcoma metastasis and chemoresistance	Directly bind to the 3’-UTR of TRIM7 mRNA	(69)
hnRNPA2/B1	Oncogene	Independent prognostic factor for overall survival		(63)
ELAVL1	Oncogene	Promote osteosarcoma growth, migration, and colony formation	DRG1	(66)

### Dysregulation of m^6^A Erasers in Osteosarcoma

The m^6^A erasers, FTO and ALKBH5, are distributed in both the nucleus and cytoplasm (63). The effect of ALKBH5 in osteosarcoma remains controversial. Chen et al. (71) revealed that ALKBH5 can promote osteosarcoma cell proliferation and tumor growth by decreasing the m^6^A modification of plasmacytoma variant translocation 1 (PVT1), subsequently impairing the binding of reader protein YTHDF2 in PVT1. However, Yuan et al. (72) showed that ALKBH5 can epigenetically silence pre-miR-181b-1/YAP signaling axis, and thus, suppress tumor progression in osteosarcoma. In addition, upregulation of ALKBH5 expression also contributes to chemoresistance and predicts worse metastasis-free survival in patients with osteosarcoma (70). The regulatory mechanism is shown in **Table 1** and **Figure 3**.

### Dysregulation of m^6^A Readers in Osteosarcoma

The subcellular location of m^6^A readers in osteosarcoma cells is relatively complicated. YTHDF1 and YTHDF2 are mainly distributed in the cytoplasm. YTHDC1 is mainly located in the nucleus, while YTHDC2 is uniformly distributed in the nucleus and cytoplasm. Additionally, HNRNPC and HNRNPA2B1 are found only in the nucleus (63). YTHDF2 can directly bind to the 3’-UTR of TRIM7 mRNA and negatively regulate the expression of TRIM7 in osteosarcoma HOS and MG63 cells (69). ELAVL1 (also known as HuR) is a recently discovered m^6^A reader, which is located in the nucleus. It has been reported to regulate the stability of DRG1 mRNA. Silencing ELAVL1 inhibits osteosarcoma progression by decreasing the expression of *DRG1* in an m^6^A-dependent pattern (66). These processes have been summarized in **Table 1** and **Figure 3**.

## m^6^A and Osteosarcoma-Related Diseases

### m^6^A and Bone Disease

As an essential component of epigenetic regulation, m^6^A modification also has inextricable link with other bone diseases, such as intervertebral disc degeneration (IDD), osteoarthritis, and osteoporosis (73). In IDD, the microarray results showed that most of the dysregulated RNA was in a demethylated state. FTO and ZFP217 can demethylate LOC102555094 and activate downstream Wnt pathway, which may contribute to metabolic reprogramming of glucose metabolism in the IDD process (74). METTL14 can accelerate TNF-α-induced nucleus pulposus cell cycle arrest and senescence *via* processing miR-34a-5p (75). In osteoarthritis, METTL3 is suggested to be responsible for the development of the disease by regulating NF-κB signaling and extracellular matrix synthesis in chondrocytes (76). As for osteoporosis, Mo et al. (77) revealed that m^6^A-associated single nucleotide polymorphisms (SNPs) can affect bone mineral density (BMI). Their GWAS result showed that 138, 125, and 993 m^6^A-SNPs were associated with femoral neck, lumbar spine, and quantitative heel ultrasounds BMI respectively. Besides, METTL3 is found to be downregulated in human osteoporosis and the ovariectomized mouse model (58). The up-regulation of METTL3 in MSCs can prevent estrogen deficiency-induced osteoporosis (78).

### m^6^A and Other Cancer

Since the mechanism of m^6^A in osteosarcoma is not well understood, we presumed that the mechanism of m^6^A in other cancer may provide some clue in this regard. Based on our review, we principally summarize the following three point:

First, the dysregulation of erasers and readers has been more investigated in other cancers. In pancreatic cancer, ALKBH5 acts as a tumor suppressor by decreasing WIF-1 RNA methylation and suppressing the Wnt pathway (79). It can also prevent the progression of pancreatic cancer by regulating the posttranscriptional activation of PER1 in an m^6^A-YTHDF2-dependent manner (80). In acute myeloid leukemia (AML), YTHDF2 decreases m^6^A RNA stability and is crucial for AML initiation and propagation (81). In breast cancer, hnRNPA2B1 can directly bind to the UAGGG locus of *PFN2* mRNA to reduce its stability, and thereby, inhibit the metastasis of breast cancer (82). In light of the significant role of m^6^A erasers and readers in cancers, we look forward to more investigation about dysregulation of erasers and readers in osteosarcoma.

Second, m^6^A modification is associated with the molecular epidemiology and clinicopathology of cancer. Zeng et al. (83) performed a case-control study based on Chinese population, which showed that different SNPs of FTO were concerned with varying risk of breast cancer. For instance, presence of rs1477196, rs16953002, and TAC haplotype (rs9939609-rs1477196-rs1121980) in the FTO gene is associated with a high risk of breast cancer. Wu et al. (84) revealed that overexpression of METTL3, METTL14, FTO, and ALKBH5, and under-expression WTAP was closely related with luminal type breast cancer, while the expression level of FTO was significantly decreased in human epidermal growth factor receptor 2 (HER2) positive breast cancer. Xiao et al. (85) illustrated that detection of m^6^A combined with METTL14 and FTO expression in peripheral blood can diagnose breast cancer with a specificity of 97.4%.

Third, recent studies have found that the tumor microenvironment can induce the dysregulation of m^6^A regulators (86–88). For example, hypoxia can alter the level/activity of METTL14, ALKBH5, and YTHDF3, leading to decreased m^6^A modification in the target transcripts in breast cancer cells (86). Stress due to metabolic starvation can elevate the expression of FTO through the autophagy and NF-κB pathways (87). Inflammatory stimuli can induce YTHDF2 expression in hematopoietic stem cell (88). The bone microenvironment including mesenchymal stem cells (MSCs), hypoxia, acidic condition, chemokines and immune cells, is regarded as fertile soil for osteosarcoma (89, 90). Given this, the elucidation of m^6^A signatures and the bone microenvironment in osteosarcoma deserves further study.

## Discussion

### Clinical Implications of m^6^A for Osteosarcoma

Considering that the dysregulation of m^6^A modification has been linked to the initiation, metastasis, drug resistance, and other processes of osteosarcoma, m^6^A may bring new breakthroughs in the diagnosis and treatment of osteosarcoma.

On the one hand, m^6^A regulatory enzymes could be novel potential biomarkers for the early diagnosis and prognosis of osteosarcoma. High m^6^A methylation levels and dysregulated m^6^A enzymes always occur in patients with osteosarcoma. Based on two large-scale cohorts, HNRNPA2B1, HNRNPC, RBM15, YTHDF1, and YTHDC1 expression levels are upregulated in osteosarcoma tissues. Among them, HNRNPA2B1 was suggested to be an independent prognostic risk factor in patients with osteosarcoma and predict poor survival rates (63). On the other hand, m^6^A may also serve as a novel therapeutic target in osteosarcoma. M^6^A modification is pivotal in almost all pathophysiological processes of osteosarcoma, including tumorigenesis, invasion, and metastasis (64, 68, 69, 71). The small molecule inhibitor of m^6^A regulators has been regarded as a kind of potential anti-cancer agent (91, 92). Currently, several FTO and ALKBH5 inhibitors have been successfully identified, including N-oxalylglycine, entacapone, and meclofenamic acid (MA) (93–95). Furthermore, *in vitro* experiments have shown that these inhibitors can inhibit tumor growth (96). Encouragingly, entacapone and MA are already in the early phases of clinical trials for patients with late-stage cancer (97). Additionally, m^6^A also plays a key role in the resistance to chemotherapy of osteosarcoma. METTL3 and ALKBH5 expression levels are upregulated in doxorubicin-resistant osteosarcoma cells, while METTL14 expression is downregulated (70). YTHDF2 knockdown can significantly increase the expression of TRIM7 and cause resistance to doxorubicin and methotrexate treatment (69). Therefore, targeting dysregulated m^6^A enzymes represents an attractive strategy for cancer therapy. It can not only directly inhibit tumor growth but also sensitize cancer cells to anti-cancer agents.

Immunotherapy has emerged as a promising treatment modality that largely expands the therapeutic regimen for cancer (98). Bone is characterized as a highly specialized immune environment, and some immune-related factors are frequently dysregulated in osteosarcoma (99). Overall, the expression level of tumor-associated macrophages is reduced in metastatic osteosarcoma, while tumor-infiltrating lymphocytes appear to be associated with enhanced metastases in osteosarcoma (100). Programmed death ligand-1, one of the most effective immune checkpoint modulators, is expressed in approximately 25% of primary osteosarcoma tumors and is associated with poor prognosis (100, 101). However, immune checkpoint inhibitors are less effective in treating osteosarcoma (102). In the SARC028 trial, only 1 of the 22 patients with osteosarcoma, who received pembrolizumab (an anti-programmed death 1 (PD-1) antibody) showed a good response (103). In the PEMBROSARC study, 17 patients with advanced osteosarcoma received a combination of pembrolizumab and cyclophosphamide, and only 2 patients had significant clinical benefits (104). Interestingly, more studies have indicated that m^6^A regulators play an essential role in host immunity and may contribute to anticancer immunotherapy (105, 106). For instance, Han et al. (105) found that the m^6^A reader, YTHDF1, can promote the translation of mRNAs encoding lysosomal proteases. Downregulation of YTHDF1 results in the reduction of antigen cross-presentation and alleviation of the cytotoxic lymphocyte response against tumor antigens in dendritic cells, thereby enhancing the effectiveness of the PD-1 blockade therapy. Similarly, the m^6^A eraser, ALKBH5, can regulate the lactate content, tumor-infiltrating regulatory T cells, and myeloid-derived suppressor cell accumulation in the tumor microenvironment. In mouse melanoma, depleting ALKBH5 improved the efficacy of anti–PD-1 therapy and had survival benefits (106). In view of the promising effect of immunotherapy in cancers and the close association of m^6^A modification with immune response, there are reasons to believe that combining anticancer immunotherapy with m^6^A signatures may pave a way to improve the therapeutic effects of osteosarcoma.

### Conclusions and Future Perspective

M^6^A modification has emerged as an indispensable factor that accounts for tumor initiation and progression in osteosarcoma. The function of m^6^A modification is just like that of a “double-edged sword”, by which it can either accelerate or inhibit the progression of osteosarcoma *via* different modes. Undoubtedly, the advent of m^6^A regulation has provided new insight into the molecular mechanism of osteosarcoma and will potentially help develop new more effective therapies.

However, a full understanding of the mechanism underlying m^6^A modification is still in its infancy, several knowledge gaps remain. First, existing studies on m^6^A in osteosarcoma mainly focused on writers; the dysregulation of m^6^A erasers and readers in osteosarcoma require further study. Second, although m^6^A is considered a potential biomarker for the diagnosis and prognosis of osteosarcoma, only few studies have yet elucidated the relationship between m^6^A-related factors and the molecular epidemiology as well as clinicopathology of osteosarcoma. Third, the clinical guidance of sequencing data of small osteosarcoma samples is limited due to the high genomic heterogeneity of this disease. Therefore, a large sample of sequencing database for m^6^A-related factors and osteosarcoma is warranted. Fourth, researchers have noted the potential of m^6^A as a therapeutic target for osteosarcoma, but few studies have focused on the application of potent and specific drugs that target m^6^A enzymes in osteosarcoma. In addition, compared with other cancer, osteosarcoma has its own biological and clinical features, such as the close connection with bone microenvironment and poor immunotherapy effect. Hence, combining m^6^A factors with tumor microenvironment and anticancer immunotherapy in osteosarcoma must be explored.

## Author Contributions

YZ, YYW, and LY conceived and designed the research. YZ, YYW, and ST searched the literature and analyzed the data. YZ and YYW wrote the manuscript and created the figures. YXW, PL, MS, and BH reviewed and made significant revisions to the manuscript. YZ, YYW, and LY contributed equally to this work as the first authors. All authors contributed to the article and approved the submitted version.

## Funding

This work was supported by the Nature Science Foundation of Zhejiang Province (LQ19H060002, LQ19H160041 and LY18H060004) and the Medical and Health Science and Technology Project of Zhejiang Province (2020KY143).

## Conflict of Interest

The authors declare that the research was conducted in the absence of any commercial or financial relationships that could be construed as a potential conflict of interest.
